# Differences in Neuropathogenesis of Encephalitic California Serogroup Viruses

**DOI:** 10.3201/eid2504.181016

**Published:** 2019-04

**Authors:** Alyssa B. Evans, Clayton W. Winkler, Karin E. Peterson

**Affiliations:** National Institute of Allergy and Infectious Diseases, National Institutes of Health, Hamilton, Montana, USA

**Keywords:** orthobunyaviruses, brain, pathogenicity, virus replication, neurons, encephalitis, neuropathogenesis, California serogroup viruses, La Crosse virus, snowshoe hare virus, Tahyna virus, Jamestown Canyon virus, Inkoo virus, viruses, vector-borne infections, mosquitoborne viruses, mouse model, C57BL/6, United States

## Abstract

The California serogroup of orthobunyaviruses comprises a group of mosquitoborne viruses, including La Crosse (LACV), snowshoe hare (SSHV), Tahyna (TAHV), Jamestown Canyon (JCV), and Inkoo (INKV) viruses, that cause neurologic disease in humans of differing ages with varying incidences. To determine how the pathogenesis of these viruses differs, we compared their ability to induce disease in mice and replicate and induce cell death in vitro. In mice, LACV, TAHV, and SSHV induced neurologic disease after intraperitoneal and intranasal inoculation, and JCV induced disease only after intranasal inoculation. INKV rarely induced disease, which correlated with less viral antigen in the brain than the other viruses. In vitro, all viruses replicated to high titers; however, LACV, SSHV, and TAHV induced high cell death, whereas JCV and INKV did not. Results demonstrated that CSG viruses differ in neuropathogenesis in vitro and in vivo, which correlates with the differences in pathogenesis reported in humans.

The California serogroup (CSG) of orthobunyaviruses comprises a large group of closely related mosquitoborne, trisegmented, negative-sense RNA viruses in the family *Peribunyaviridae* of the order Bunyavirales. La Crosse virus (LACV), snowshoe hare virus (SSHV), Tahyna virus (TAHV), Jamestown Canyon virus (JCV), and Inkoo virus (INKV) are members of the CSG that have been reported to cause neurologic disease in humans. LACV, SSHV, TAHV, and INKV primarily cause neuroinvasive disease in children; however, the incidence differs for each virus (*1*–*3*). LACV is the leading cause of pediatric viral encephalitis in the United States, responsible for ≈50–100 reported cases per year. SSHV and TAHV only cause several reported cases of neuroinvasive disease annually (*1*,*2*,*4*–*6*), and TAHV disease most often manifests as influenza-like symptoms and only rarely leads to encephalitis (*2*,*7*). INKV has caused the fewest cases of human disease. However, several confirmed neuroinvasive cases have occurred in Finland, and children had more severe disease than adults (3)*.* In contrast, JCV appears to preferentially cause neuroinvasive disease in adults, and several cases occur every year in the United States and Canada (*1*,*8*). Because the number of reported cases increased substantially in the 2010s, JCV is considered a potentially emerging arboviral disease (*9*).

The number of actual cases caused by these CSG viruses is likely underreported because of the difficulty of diagnosing these viral infections. Patients who do not seek medical treatment and those with less severe disease might not be included in case reports. All 5 viruses have high reported seroprevalence rates in endemic regions: ≈1%–27% for LACV, JCV, and SSHV; ≈24%–51% for INKV; and up to 80% for TAHV (*1*,*2*,*10*–*12*).

These CSG viruses have differing but overlapping geographic distributions (Figure 1, panel A). LACV, JCV, and SSHV are all found in the United States; JCV and SSHV extend into Canada, and SSHV extends into Russia (*1*,*2*,*4*,*8*,*10*,*13*–*28*). Although TAHV and INKV are primarily found in Europe, TAHV extends into Africa and Asia, and INKV is limited to northern Europe and Russia (*2*,*3*,*12*,*29*,*34*–*36*)*.* These CSG viruses use a variety of mosquito vectors, primarily in the *Aedes* and *Ochlerotatus* genera, and mammalian host species, including small rodents (SSHV, LACV, and TAHV), hares (SSHV, TAHV, and INKV), and deer (JCV) (Figure 1, panel B) (*1*,*2*,*12*,*22*,*29*,*31*–*33*).

**Figure 1:**
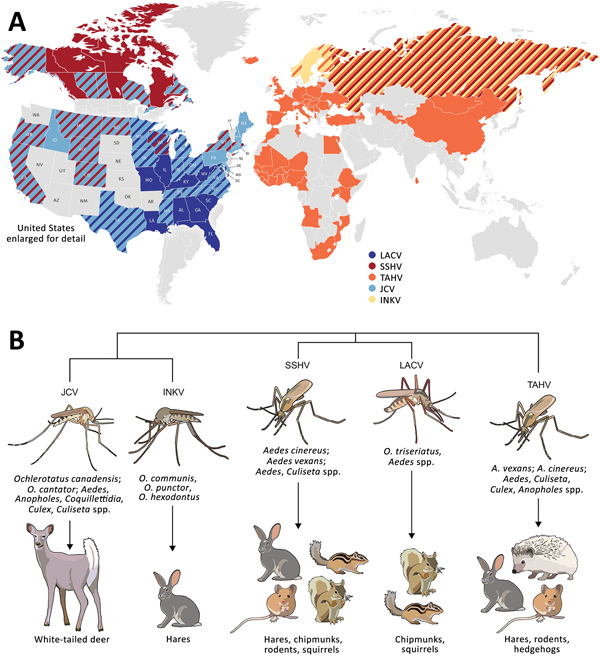
Global distribution, phylogenetic relationship, and vectors and hosts of the 5 California serogroup (CSG) viruses included in study of neuropathogenesis. A) These viruses are found across the globe, primarily throughout North America, Europe, Asia, and Africa (*1*,*4*,*8*,*10*,*12*–*29*). Several of these viruses have overlapping regions of distribution (as indicated by diagonal patterns), including in the United States, where LACV, SSHV, and JCV are all present, and Europe, where TAHV and INKV are present. States and countries indicated have evidence of these viruses from reported human cases, serologic surveys of humans and animals, or isolation of virus from mosquitoes. B) Within these closely related CSG viruses, JCV and INKV are the most closely related, followed by LACV and SSHV, and then TAHV (*30*). The CSG viruses use a variety of mosquito vectors, primarily in the *Aedes* and *Ochlerotatus* genera. Listed are the most prominent vectors and additional genera the virus has been found in. Mammals implicated as reservoir or amplifying hosts are listed for each virus; some hosts are shared among several CSG viruses (*1*,*2*,*12*,*22*,*31*–*33*). INKV, Inkoo virus; JCV, Jamestown Canyon virus; LACV, La Crosse virus; SSHV, snowshoe hare virus; TAHV, Tahyna virus.

Despite their differing and widespread geographical distributions, the CSG viruses are genetically closely related. The large segment encodes the viral RNA-dependent RNA polymerase, the medium segment encodes 2 envelope glycoproteins and a nonstructural protein, and the small segment encodes the nucleocapsid and a second nonstructural protein (NSs), which in LACV is a type I interferon antagonist (*37*,*38*). Across all 3 segments, JCV and INKV are the most closely related to each other, with 84%–92.4% nucleotide identity, followed by LACV and SSHV with 79.4%–89.1% nucleotide identity (*30*). TAHV has ≈72.5%–84.7% nucleotide identity with LACV and SSHV and 69.2%–80.3% with JCV and INKV (*30*).

In mouse studies of LACV, weanling C57BL/6 mice were susceptible to neurologic disease after intraperitoneal inoculation, but adult mice were not, demonstrating age-dependent susceptibility to disease that mimics clinical disease in humans (*39*). Certain strains of TAHV, but not JCV, have been shown to cause neurologic disease in weanling Swiss Webster mice after intraperitoneal inoculation, although both viruses were capable of replicating in the brain after intracranial inoculation (*40*,*41*). In these studies, only weanling mice were used, and thus, age-dependent susceptibility was not evaluated.

Determining how LACV, SSHV, TAHV, JCV, and INKV differ in pathogenesis in animal and cell culture models could help explain their differing disease outcomes in humans. However, a direct comparison between these viruses in their ability to invade the central nervous system (CNS) and induce neuronal damage has not been investigated. In this study, we investigated age-related differences in pathogenesis of LACV, SSHV, TAHV, JCV, and INKV. We examined the ability of these viruses to enter the CNS and cause disease in mice and their ability to replicate in neurons and induce cell death in vitro.

## Materials and Methods

### Cells and Viruses

All cell culture reagents were from Gibco (http://www.biosciences.ie/gibco) unless otherwise specified. We maintained Vero cells in Dulbecco modified Eagle medium supplemented with 10% fetal bovine serum (Atlas Biologicals, https://atlasbio.com) and 1% penicillin/streptomycin solution; C6/36 cells in minimum essential medium supplemented with 10% fetal bovine serum, 2 mM glutamine, 1× nonessential amino acids, and 1% penicillin/streptomycin; the neuroblastoma cell line SH-SY5Y (American Type Culture Collection [ATCC], https://www.atcc.org) in a 1:1 ratio of Eagle minimum essential medium (ATCC) and F-12K (ATCC) supplemented with 10% fetal bovine serum and 1% penicillin/streptomycin; and H9 human embryonic stem cell–derived human neural stem cells (hNSCs; Gibco, https://www.fishersci.com) in KnockOut DMEM/F-12 supplemented with 1× GlutaMAX-I Supplement, 20 ng/mL basic fibroblast growth factor, 20 ng/mL epidermal growth factor, 2% StemPro neural supplement, and 1% penicillin/streptomycin. We seeded hNSCs on plates or flasks treated with 20 μg/mL fibronectin in Dulbecco phosphate-buffered saline for 1 h at 37°C. We passaged stocks of LACV (human 1978 strain), JCV (strain 61V2235), TAHV (strain 92 Bardos), SSHV (1976), and INKV (SW AR 83-161), all kindly provided by Stephen Whitehead (Laboratory of Infectious Diseases, National Institute of Allergy and Infectious Diseases, National Institutes of Health, Bethesda, MD, USA), in Vero or C6/36 cells up to 3 times.

### Inoculation of Mice

All mouse experiments were approved by the Rocky Mountain Laboratories Animal Care and Use Committee (Hamilton, Montana, USA) and performed in accordance with National Institutes of Health guidelines under protocol 2016-061-E. We used C57BL/6 mice for all experiments.

We inoculated weanlings at 21–23 days of age, adults at 6–8 weeks of age, and aged mice at 19–34 weeks of age. We diluted the viruses in phosphate-buffered saline (PBS). For intraperitoneal inoculations, we injected mice with 10^5^ or 10^3^ PFU of virus in a volume of 200 μL. For intranasal inoculations, we inoculated mice with 10^4^ or 10^2^ PFU of virus in a volume of 20 μL. We anesthetized mice with isoflurane before inoculation. After inoculation, we checked mice twice daily for clinical signs of neurologic disease, which primarily included ataxia, circling, limb paralysis and weakness, twitching, and seizures. Mice displaying signs of neurologic disease were perfused transcardially with 100 U/mL heparin saline before removal of tissues. We humanely euthanized mice that did not display any signs of neurologic disease by 30 days postinoculation (dpi).

### RNA Isolation and Quantitative Reverse Transcription PCR

For RNA analysis, we flash-froze spleens and brain tissue from infected mice in liquid nitrogen, then stored them at −80°C. We performed mRNA analysis as previously described (*42*)*.* We amplified viral RNA from mouse tissues using the following virus-specific primers: LACV forward (5′-ATTCTACCCGCTGACCATTG-3′) and reverse (5′-GTGAGAGTGCCATAGCGTTG-3′), SSHV forward (5′-AGCATGATCAAAACGGAGGC-3′) and reverse (5′-CATGCCAATCAGACACCAGC-3′), TAHV forward (5′-AGGTCCTACATTGCCGTTCA-3′) and reverse (5′-TGGTCTACAGGTGCTAGCTC-3′), JCV forward (5′-′TATGGTTCCCCGGTAGTGTG-3′) and reverse (5′-TAACATGGTGCTTCTCGTGC-3′), and INKV forward (5′-AGTCCAAGATAAAGCCCCAGA-3′) and reverse (5′-TCATGTTAGCCTGGCATCCA-3′). We subjected primer sequences to BLAST analysis (https://blast.ncbi.nlm.nih.gov/Blast.cgi) and tested each primer set on RNA from brains of mice infected with the 5 CSG viruses that had neurologic disease to verify virus-specific amplification by each primer set.

### Immunohistochemistry

For immunohistochemistry studies, we transcardially perfused mice, removed tissues, and placed them in 10% neutral buffered formalin. After fixation, we cryoprotected tissues in 30% sucrose in PBS, embedded them in Tissue-Tek O.C.T. Compound (Sakura, https://www.sakura.eu) and froze them on dry ice; then, 10-μm sections were cut on a cryostat and mounted on slides. We washed sections with PBS and blocked for 30 minutes in blocking buffer (PBS with 5% normal donkey serum, 0.1% triton-X, and 0.3 M glycine). We diluted the primary antibody against viral antigens (in-house polyclonal antibody raised in rabbits in response to LACV infection, 1:100) and microtubule-associated protein 2 (mouse monoclonal antibody MAB3418, 1:200; Millipore, http://www.emdmillipore.com) in blocking buffer, applied these solutions to tissues, and incubated them overnight at 4°C. We washed tissue sections with PBS and incubated for 1 hour at room temperature with donkey anti-rabbit Alexa Fluor 647 (1:1,000, Life Technologies, https://www.thermofisher.com) and donkey anti-mouse Alexa Fluor 594 (1:500, Life Technologies). After washing sections again with PBS, we stained with Hoechst (1:1,000) and mounted on slides with ProLong Gold (Invitrogen, https://www.thermofisher.com). We imaged entire brain sections using the Zeiss Axio Scan.Z1 (https://www.zeiss.com) with the 40× objective lens. We acquired high-resolution images on the Zeiss 710 laser scanning microscope with the 63× objective and processed images in Imaris version 8.4.1 (https://imaris.oxinst.com) or Fiji (http://fiji.sc).

### Cytotoxicity Assays

We coated 96-well plates with fibronectin as previously described and seeded 5 × 10^4^ SH-SY5Y cells or 2 × 10^4^ hNSCs per well. The next day, cells were inoculated with each virus in triplicate at multiplicities of infection (MOIs) of 0.1 and 0.01 for SH-SY5Y cells and 0.01 and 0.001 for hNSCs. We added Cytotox Green (Essen Bioscience, https://www.essenbioscience.com) for a final well volume of 100 μL and a final concentration of 250 nM. Cells were then imaged with an IncuCyte (Essen Bioscience) by taking 3 images per well with the 20× objective every 3 hours during a 97-hour time course. We measured confluence and fluorescent intensity using IncuCyte S3 software. We performed all statistical analyses using Prism 7.0c (https://www.graphpad.com).

### Replication Kinetics

We plated and inoculated cells as described in the previous section, except that plates seeded with SH-SY5Y cells were not treated with fibronectin. We collected supernatants at 1, 6, 12, 24, 48, 72, and 96 hours postinoculation. We determined virus titers of supernatants by plaque assay using Vero cells as described previously (*43*). We counted LACV, TAHV, and JCV plaques at 5 dpi and SSHV and INKV plaques at 3 dpi. We performed all statistical analyses using Prism 7.0c (https://www.graphpad.com).

## Results

### Assessment of Neuroinvasive Disease in Mice after Intraperitoneal Inoculation with CSG Viruses

**Figure 2:**
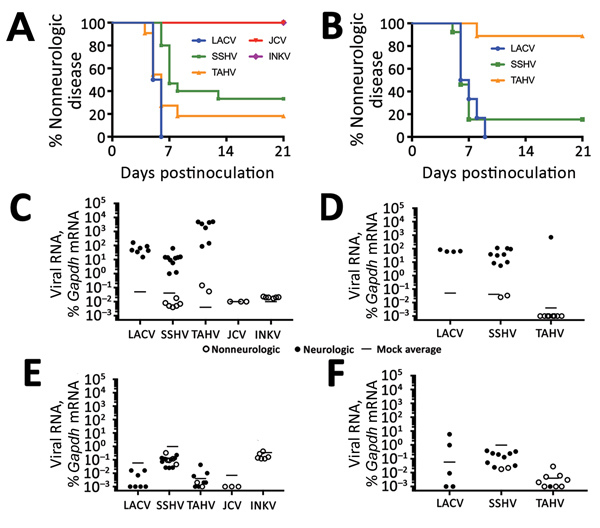
Neuroinvasiveness of California serogroup (CSG) viruses in weanling C57BL/6 mice after intraperitoneal inoculation in study of neuropathogenesis. We inoculated 5–15 mice per group with 10^5^ PFU of each virus (A) and 6–13 mice per group with 10^3^ PFU of LACV, SSHV, or TAHV (B). Brains and spleens of mice were collected at the experimental endpoint and evaluated for viral RNA by quantitative reverse transcription PCR with virus-specific primers. The average of 3 mock controls is reported for each primer set. The viral RNA level in each sample was calculated as the difference in the percentage in cycle threshold (C_t_): C_t_ for Gapdh mRNA minus C_t_ for viral mRNA. Viral RNA was plotted as the percentage of gene expression relative to that of the *Gapdh* gene. Viral RNA in brains of mice inoculated with 10^5^ PFU of each virus (C) or 10^3^ PFU of LACV, SSHV, and TAHV (D). Viral RNA in spleens of mice inoculated with 10^5^ PFU of each virus (E) or 10^3^ PFU of LACV, SSHV, and TAHV (F). Gapdh, glyceraldehyde 3-phosphate dehydrogenase; INKV, Inkoo virus; JCV, Jamestown Canyon virus; LACV, La Crosse virus; SSHV, snowshoe hare virus; TAHV, Tahyna virus.

To determine if these 5 CSG viruses differed in their ability to enter the CNS and cause neurologic disease (i.e., neuroinvasive disease), C57BL/6 mice were inoculated intraperitoneally with a high dose (10^5^ PFU) of each virus. Because previous studies of LACV infection showed clear age-dependent differences in disease susceptibility between weanling and adult mice, we inoculated weanling, adult, and aged mice with each virus and followed them for the development of clinical signs of neurologic disease. None of the viruses induced neuroinvasive disease in adult or aged mice after intraperitoneal inoculation (data not shown). However, neuroinvasive disease developed by 5–6 dpi in all weanling mice inoculated with 10^5^ PFU of LACV (Figure 2, panel A), consistent with previous reports (*39*,*44*). SSHV induced neuroinvasive disease in ≈70% of weanling mice and TAHV in ≈80% of weanling mice. Neurologic signs were primarily observed at 6–7 dpi for SSHV and 5–6 dpi for TAHV. Neurologic signs were similar for LACV, SSHV, and TAHV and included ataxia, circling, limb paralysis and weakness, twitching, and seizures. JCV and INKV did not induce neuroinvasive disease in any weanling mice.

In previous studies with LACV, nearly 100% of weanling mice were susceptible to neuroinvasive disease after intraperitoneal inoculation at a dose of 10^3^ PFU (*39*). To determine if TAHV and SSHV also maintain their pathogenicity at this low dose, we inoculated weanling mice intraperitoneally with 10^3^ PFU of LACV, SSHV, or TAHV. JCV and INKV were not included in this experiment because they did not induce neuroinvasive disease at the high dose. Consistent with previous studies, 10^3^ PFU of LACV caused disease in 100% of mice but with a slight delay in the onset of neurologic signs compared with the 10^5^ PFU dose (Figure 2, panel B). At the low dose, SSHV induced disease in a similar percentage of mice as the high dose, whereas TAHV caused disease in only 1 of 9 mice at the low dose (Figure 2, panel B).

All weanling mice with neurologic disease had detectable viral RNA in the brain, whereas weanling, adult, and aged mice without neurologic disease had no detectable virus in the brain compared with mock controls (Figure 2, panels C, D; data not shown). Regardless of disease outcome, mice had little to no detectable viral RNA in the spleen (Figure 2, panels E, F; data not shown), suggesting clearance of virus from the periphery.

Overall, these results indicate that LACV, SSHV, and TAHV can induce neuroinvasive disease in weanling mice after intraperitoneal inoculation, whereas JCV and INKV cannot. None of the CSG viruses caused neurologic signs in adult or aged mice after intraperitoneal inoculation, suggesting an age-related susceptibility of mice to LACV-, SSHV-, and TAHV-induced neuroinvasive disease.

### Assessment of Neurovirulence of CSG Viruses after Intranasal Inoculation of Mice

**Figure 3:**
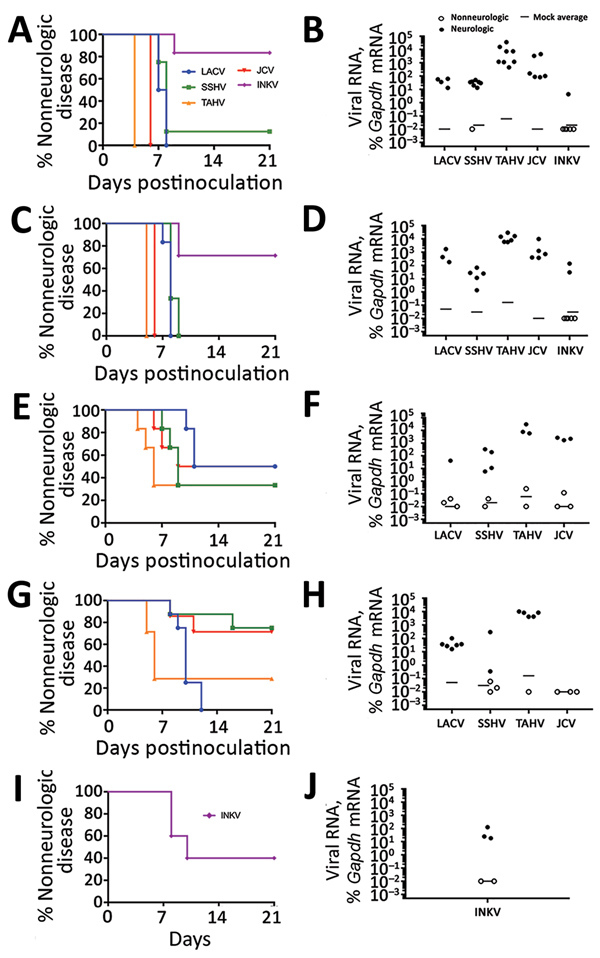
Neurovirulence of California serogroup (CSG) viruses in adult, aged, and weanling mice after intranasal inoculation in study of neuropathogenesis of encephalitic CSG viruses. Groups of adult (A, B) and aged (C, D) mice (6–8 mice per group) were inoculated with 10^4^ PFU of each virus; groups of adult (E, F) and aged (G, H) mice (6–8 mice per group) were inoculated with 10^2^ PFU of LACV, SSHV, TAHV, and JCV; and 5 weanling mice were inoculated with 10^4^ PFU of INKV (I, J). E, G) Survival rate differences between adult and aged mice infected with 10^2^ PFU of virus were calculated for each virus by using the Gehan-Breslow-Wilcoxon test. LACV was the only virus with a significant difference (p = 0.035). B, D, F, H, J) Viral RNA in mouse brains was analyzed by quantitative reverse transcription PCR with virus-specific primers. The average of 3 mock controls is reported for each primer set. The viral RNA level in each sample was calculated as the difference in the percentage in cycle threshold (C_t_): C_t_ for Gapdh mRNA minus C_t_ for viral mRNA. Viral RNA was plotted as the percentage of gene expression relative to that of the *Gapdh* gene. Gapdh, glyceraldehyde 3-phosphate dehydrogenase; INKV, Inkoo virus; JCV, Jamestown Canyon virus; LACV, La Crosse virus; SSHV, snowshoe hare virus; TAHV, Tahyna virus.

The inability of a virus to cause neuroinvasive disease after intraperitoneal inoculation could be due to a lack of virus replication in the periphery or an inability of the virus to gain access to the CNS. These barriers can be bypassed by intracranial or intranasal inoculation. For example, LACV is not neuroinvasive in adult mice after intraperitoneal inoculation but does cause disease in adult mice when inoculated intracranially or intranasally (*39*,*44*,*45*). Because none of these CSG viruses caused disease in older mice after intraperitoneal inoculation, adult and aged mice were inoculated intranasally with each virus to determine if they were neurovirulent (i.e., could replicate in the brain) and neuropathogenic (i.e., could cause disease in the brain) after a more direct inoculation route and to determine if neuropathogenesis was restricted by age. Because of the low stock concentration of some viruses, we could not inoculate mice with 10^5^ PFU intranasally; therefore, we used a high dose of 10^4^ PFU and a low dose of 10^2^ PFU. At a dose of 10^4^ PFU per mouse, all viruses except INKV induced neurologic disease in nearly all adult and aged mice (Figure 3, panels A, C). INKV was less neuropathogenic than the other CSG viruses, inducing disease in only 1 of 6 adult mice and 2 of 7 aged mice. Compared with the high dose, the low dose induced neurologic disease in a reduced percentage of mice for all viruses, except LACV in aged mice (Figure 3, panels E, G). Viral RNA was readily detectable in the brains (Figure 3, panels B, D, F, H) but not spleens (data not shown) of all mice with neurologic disease.

Because INKV induced limited disease in adult mice, we inoculated weanling mice intranasally with INKV to determine if neuropathogenicity was age dependent. Neurologic disease developed in 3 (60%) of 5 mice and was associated with viral RNA in the brain (Figure 3, panels I, J). Thus, with a high dose and direct intranasal route to the CNS, INKV was more neuropathogenic in young mice than in adults (Figure 3, panels B, J).

**Figure 4:**
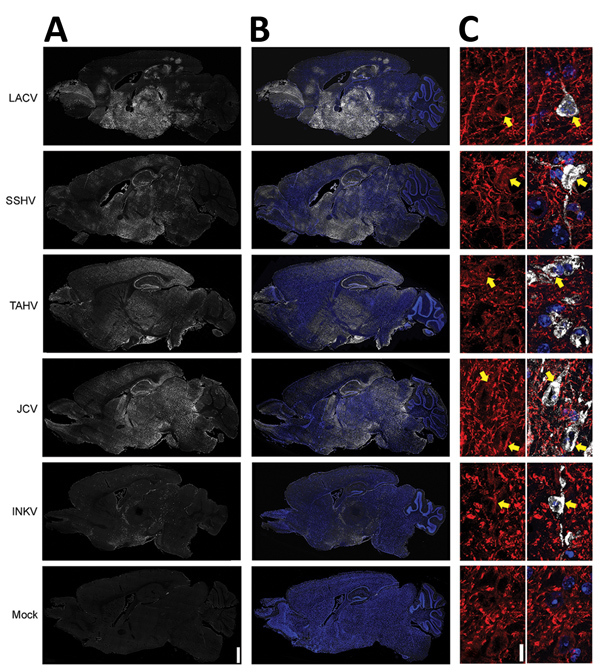
Viral antigen in brains of adult and aged mice exhibiting neurologic disease after intranasal inoculation of 10^4^ PFU of California serogroup (CSG) viruses in study of neuropathogenesis. We evaluated >4 brains from mice infected with each CSG virus, except INKV (where only 3 brains from mice with neurologic disease were available), for viral immunoreactivity. A, B) Representative images showing distribution of virus (white) and virus merged with Hoechst nuclear stain (blue) via full-section brain scans. Scale bar indicates 1 mm. C) Maximum intensity projections of 5-μm confocal z-stacks (original magnification ×63) of brains of mice infected with the indicated CSG virus, labeled for virus (white) and the neuronal marker microtubule-associated protein 2 (MAP2; red). Left panels demonstrate MAP2 staining alone, and right panels are overlays of virus, MAP2, and Hoechst nuclear stain (blue). Yellow arrows indicate the soma of neurons where both viral and MAP2 immunoreactivity are found. Scale bar indicates 10 μm. INKV, Inkoo virus; JCV, Jamestown Canyon virus; LACV, La Crosse virus; SSHV, snowshoe hare virus; TAHV, Tahyna virus.

Next, we examined the distribution of the CSG viruses in the brain using immunohistochemistry. In mice inoculated intranasally with 10^4^ PFU of LACV, SSHV, TAHV, or JCV that had neurologic disease, virus was widespread throughout the brains regardless of the day after inoculation or severity of neurologic disease (Figure 4, panels A, B). In contrast, the brains of adults and aged mice displaying neurologic disease after intranasal inoculation of INKV showed only small, sporadic patches of virus (Figure 4, panels A, B). All viruses co-localized with the neuronal marker microtubule-associated protein 2, indicating that all CSG viruses primarily infected neurons within the CNS (Figure 4, panel C). Overall, these results demonstrate that LACV, SSHV, TAHV, and JCV are neurovirulent in mice when inoculated intranasally, whereas INKV appears to be less neurovirulent and infects fewer neurons within the CNS.

### Ability of CSG Viruses to Replicate in and Kill Neurons in vitro

**Figure 5:**
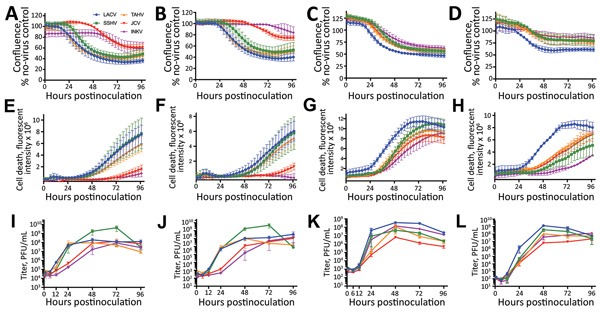
Cytotoxicity and viral replication kinetics assays of California serogroup (CSG) viruses in SH-SY5Y cells and human neural stem cells (hNSCs) for up to 96 hours postinoculation in study of neuropathogenesis. SH-SY5Y cells were infected at a multiplicity of infection of 0.1 (A, E, I) or 0.01 (B, F, J), and hNSCs were infected at a multiplicity of infection of 0.01 (C, G, K) or 0.001 (D, H, L). A–D) Confluence was measured over time on the IncuCyte (Essen Bioscience, https://www.essenbioscience.com) as the percentage of the image covered by cells. Graphs show the percentage of confluence compared with that of uninfected control wells. E–H) Cell death was measured over time with the IncuCyte and reported as the total integrated fluorescent intensity of the Cytotox Green (Essen Bioscience) reagent. I–L) Supernatants were harvested from SH-SY5Y cells and hNSCs at 1, 6, 12, 24, 48, 72, and 96 hours postinfection and titered on Vero cells by plaque assay. All error bars indicate SEM. PFU, plaque-forming units.

The differences in pathogenicity among the CSG viruses observed in mice and humans could be related to differences in their ability to infect and kill neurons. Therefore, we analyzed the ability of these viruses to replicate in and kill neurons using the human neuroblastoma cell line SH-SY5Y and hNSCs. We inoculated cells with each virus at MOIs predetermined to provide a sufficient time frame to measure virus replication before the onset of substantial cell death with LACV (MOIs of 0.1 and 0.01 for SH-SY5Y cells and MOIs of 0.01 and 0.001 for hNSCs). In SH-SY5Y cells, LACV, SSHV, and TAHV induced substantial cell death at both MOIs, whereas JCV and INKV induced little cell death (Figure 5, panels A, B, E, F). However, JCV and INKV replicated to similar titers as LACV (Table 1), albeit with a delay in replication (Table 1; Figure 5, panels I, J). In hNSCs, all 5 viruses induced cell death at both MOIs (Figure 5, panels C, D, G, H); however, LACV had a more pronounced cell death rate than the other viruses, indicating that LACV may be more neurotoxic to hNSCs than the other 4 CSG viruses, most notably INKV (Table 2; Figure 5, panels G, H). All viruses rapidly replicated to high titers at both MOIs in hNSCs; however, JCV appeared to replicate slower and reached a lower peak titer than the other CSG viruses (Table 2; Figure 5, panels K, L). All CSG viruses had significantly slower rates of growth in hNSCs with 1 or both MOIs compared with LACV (Table 2).

**Table 1:** Cell death and replication kinetics of 5 encephalitic California serogroup viruses in SH-SY5Y cells*

Virus	MOI 0.1							MOI 0.01					
Difference in cell death†			Virus replication			Difference in cell death†			Virus replication		
Range, hpi	p value range		Replication rate p value‡	Peak titer, PFU/mL	hpi§	Range, hpi	p value range		Replication rate p value‡	Peak titer, PFU/mL	hpi§
LACV	Ref	Ref		Ref	1.93 × 10^8^	48		Ref	Ref		Ref	3.51 × 10^8^	96
SSHV	NS	NS		**0.041**	4.36 × 10^9^	72		NS	NS		**0.038**	4.82 × 10^9^	72
TAHV	NS	NS		0.110	9.13 × 10^7^	48		NS	NS		0.995	1.05 × 10^8^	48
JCV	61–96	0.047–0.0001		**0.033**	1.33 × 10^8^	72		67–96	0.022–0.0001		**0.034**	1.09 × 10^8^	96
INKV	64–96	0.024–0.0001		**0.002**	8.72 × 10^7^	72		67–96	0.023–0.0001		**0.023**	1.55 × 10^8^	96

*All statistical comparisons were made with LACV used as the reference. hpi, hour postinoculation; INKV, Inkoo virus; JCV, Jamestown Canyon virus; LACV, La Crosse virus; MOI, multiplicity of infection; NS, not significant; Ref, referent; SSHV, snowshoe hare virus; TAHV, Tahyna virus. †Cell death for >3 independent experiments analyzed by 2-way analysis of variance. ‡Replication rates from 3 independent experiments were analyzed via linear regression analysis of slopes during the log phase of viral growth (6–48 hpi). Significant p values (<0.05) are in boldface. §Time point at which the sampled peak titer was observed.

**Table 2:** Cell death and replication kinetics of 5 encephalitic California serogroup viruses in human neural stem cells*

Virus	MOI 0.01							MOI 0.001					
Difference in cell death†			Virus replication			Difference in cell death†			Virus replication		
Range, hpi	p value range		Replication rate p value‡	Peak titer, PFU/mL	hpi§	Range, hpi	p value range		Replication rate p value‡	Peak titer, PFU/mL	hpi§
LACV	Ref	Ref		Ref	3.63 × 10^8^	48		Ref	Ref		Ref	1.34 × 10^9^	48
SSHV	34–49	0.042–0.013		**0.018**	4.48 × 10^7^	48		49–94	0.044–0.001		0.091	3.82 × 10^8^	48
TAHV	43–61	0.043–0.025		0.050	1.16 × 10^8^	48		64–70	0.047–0.044		**0.042**	1.08 × 10^8^	48
JCV	34–73	0.027–0.003		**0.012**	6.42 × 10^6^	48		58–76	0.037–0.015		**0.033**	2.21 × 10^7^	96
INKV	34–79	0.026–0.0001		0.075	1.43 × 10^8^	48		46–96	0.045–0.0001		**0.035**	1.30 × 10^8^	96

*All statistical comparisons were made with LACV used as the reference. hpi, hour postinoculation; INKV, Inkoo virus; JCV, Jamestown Canyon virus; LACV, La Crosse virus; MOI, multiplicity of infection; NS, not significant; Ref, referent; SSHV, snowshoe hare virus; TAHV, Tahyna virus. †Cell death for >3 independent experiments analyzed by 2-way analysis of variance. ‡Replication rates from 3 independent experiments were analyzed via linear regression analysis of slopes during the log phase of viral growth (6–48 hpi). Significant p values (<0.05) are in boldface. §Time point at which the sampled peak titer was observed.

Together, these results suggest that LACV, SSHV, and TAHV are capable of replicating quickly to high titers in neurons and inducing substantial cell death. In contrast, INKV and JCV replicated more slowly but to similar titers. INKV and JCV also induced less cell death than the other CSG viruses, although the level of cell death varied by cell type. The lower cell death associated with INKV infection in vitro correlates with the low level of INKV infection within the CNS in vivo.

## Discussion

In our study, the CSG viruses differed in pathogenesis both in mice and in vitro. Overall, LACV showed the highest neuropathogenicity and neurovirulence in vitro and in vivo, whereas INKV was the least pathogenic. These results appear to be consistent with disease patterns observed in humans, with LACV reported to cause the most neuroinvasive cases, and INKV only infrequent cases (*3*,*4*,*14*)*.* Only LACV, TAHV, and SSHV were capable of causing neuroinvasive disease, a finding only observed in weanling mice. The lack of disease in adult animals is consistent with previous findings with LACV, where an age-dependent immune response in adult mice protects them from virus-induced neurologic disease (*39*,*46*).

LACV and SSHV maintained similar levels of neuroinvasiveness down to a dose of 10^3^ PFU, whereas TAHV’s ability to induce neuroinvasive disease was greatly diminished at this low dose (Figure 2, panels A, B). The difference in dose-dependent disease susceptibility suggests that TAHV might be less virulent than LACV or SSHV. The lower virulence of TAHV than LACV or SSHV correlates with the low number of TAHV-induced neuroinvasive cases in humans, despite TAHV’s larger geographic distribution and higher rates of seroprevalence than LACV (*1*,*2*,*4*)*.*

When the peripheral immune system was bypassed and adult and aged mice were inoculated intranasally, all viruses except INKV replicated extensively throughout the brain and caused neurologic disease (Figure 4). However, INKV did cause disease in a few adult and aged mice and 60% of weanling mice after intranasal inoculation, indicating INKV can be neuropathogenic under certain conditions, particularly in younger animals. These results are consistent with human case reports showing that INKV causes more severe disease in children than adults (*3*).

Less viral antigen was found in the brains of the 3 adult and aged INKV-inoculated mice that developed neurologic disease than in the brains from mice infected with the other CSG viruses (Figure 4). INKV induced less cell death than LACV in SH-SY5Y cells, suggesting that the lack of neuropathogenicity observed with INKV in vivo could be due to low levels of INKV-induced neuronal cell death. In addition, INKV also replicated slower than LACV in SH-SY5Y cells, suggesting the limited spread of INKV in the brains of mice might be due to inefficient replication of INKV in neurons. Further studies are needed to determine why INKV has a reduced ability to induce neuronal death compared with other CSG viruses.

In humans, JCV appears to be distinct from the other CSG viruses in that JCV preferentially causes neuroinvasive disease in adults rather than in children. Although we did not observe a similar age-dependent difference in neuroinvasion of JCV in mice, this virus did differ from the other CSG viruses. JCV (like INKV) was unable to invade the CNS, yet JCV (like LACV, SSHV, and TAHV) was highly neurovirulent in brains of mice when inoculated intranasally. However, the same high level of neurovirulence was not observed in vitro. In both SH-SY5Y cells and hNSCs, JCV induced less cell death and replicated slower than LACV. These findings suggest there might be underlying genetic differences or complexities in JCV pathogenesis not shared by any of the other CSGs.

Given that genetic variation exists within and between these encephalitic CSG viruses (*30*), additional differences in pathogenicity might exist for different isolates not tested in our studies. However, overall these results show that, despite being closely related, these CSG viruses differ substantially in their abilities to induce neurologic disease in mice and replicate in neurons in vitro and in vivo. Further characterization of the host and viral factors that contribute to the differences in CSG virus pathogenesis will help determine why INKV is less neurovirulent than the other CSG viruses and elucidate potential targets for CSG virus encephalitis therapies.
